# *JAK2*^V617F^-positive clonal hematopoiesis in germline *BRCA1* versus *BRCA2* mutation carriers

**DOI:** 10.1038/s41375-026-03065-3

**Published:** 2026-07-15

**Authors:** Maria Jimena Rodriguez, Jan Hauke, Mohamad Kayali, Julian Baumeister, Marcelo A. S. de Toledo, Christoph Engel, Robert Meyer, Wouter Hubens, Wolfgang Wagner, Martina Wessiepe, Rita K. Schmutzler, Kerstin Rhiem, Tim H. Brümmendorf, Miriam Elbracht, Eric Hahnen, Steffen Koschmieder

**Affiliations:** 1 Department of Hematology, Oncology, Hemostaseology, and Stem Cell Transplantation, Faculty of Medicine, RWTH Aachen University, Aachen, Germany; 2 Center for Integrated Oncology Aachen Bonn Cologne Düsseldorf (CIO ABCD), Aachen, Germany; 3 Center for Familial Breast and Ovarian Cancer, Institute for Hereditary Cancers, Center for Integrated Oncology (CIO), University of Cologne, Faculty of Medicine and University Hospital Cologne, Cologne, Germany; 4 Institute for Medical Informatics, Statistics and Epidemiology, University of Leipzig, Leipzig, Germany; 5 Center for Human Genetics and Genomic Medicine, Medical Faculty, RWTH Aachen University, Aachen, Germany; 6 Institute of Stem Cell Biology, Faculty of Medicine, RWTH Aachen University, Aachen, Germany; 7 Helmholtz-Institute for Biomedical Engineering, RWTH Aachen University, Aachen, Germany; 8 Institute for Transfusion Medicine, Faculty of Medicine, RWTH Aachen University, Aachen, Germany

**Keywords:** Cancer genetics, Myeloproliferative disease

## Introduction

Myeloproliferative neoplasms (MPN) are clonal hematopoietic stem cell disorders characterized by excessive proliferation of one or more myeloid lineages [1]. The *JAK2*^V617F^ mutation, resulting in constitutive JAK-STAT activation, represents the most frequent driver event and is a key diagnostic criterion in MPN [1]. While most cases arise sporadically, familial clustering accounts for approximately 7% of diagnoses [2], suggesting an inherited predisposition facilitating the acquisition of somatic driver mutations such as *JAK2*^V617F^.

Genes involved in DNA damage response (DDR) have emerged as plausible contributors to inherited susceptibility in myeloid neoplasms [3, 4]. In young-onset and familial MPN, we and others previously reported enrichment of deleterious germline variants affecting DDR genes, including *BRCA1*, *BRCA2*, *CHEK2*, and *ATM* [2, 5–8]. These observations support the concept that impaired DNA repair promotes genomic instability and somatic driver acquisition, underscoring the relevance of germline genetics in selected clinical contexts.

Beyond overt MPN, *JAK2*^V617F^ can be detected at low variant allele frequencies (VAF) in individuals without hematologic malignancy, including the general population and patients with cardiovascular disease, including thromboembolism [9]. Population studies report prevalence estimates of 0.1–3.1%, depending on cohort characteristics and assay sensitivity, and even low-level positivity has been associated with increased thrombotic risk, consistent with clonal hematopoiesis (CH) contributing to cardiovascular morbidity [9–11].

However, *JAK2*^V617F^ prevalence and allele burden in germline *BRCA1* or *BRCA2* mutation carriers have not been systematically assessed. Given their central yet partially distinct roles in DDR, subtype-specific analyses are of particular interest. Here, we screened *BRCA1/2* mutation carriers, non-carrier breast cancer controls, and healthy donors for *JAK2*^V617F^ using a highly sensitive quantitative PCR approach.

## Methods

Three independent cohorts were analyzed in parallel. The first two cohorts were provided by the University Hospital Cologne and included a nationwide cohort of 568 germline *BRCA1/2* mutation carriers (324 with breast cancer) and 567 *BRCA*-wildtype breast cancer patients who had undergone clinical *BRCA* testing. The third cohort comprised 356 healthy blood donors from the Transfusion Department at RWTH Aachen University Hospital, without known malignancy at the time of blood collection; these donors were not systematically tested for germline *BRCA* variants. Peripheral blood was collected at a single time point each. Clinical characteristics are summarized in Table 1 and Supplementary Table 1. All participants provided written informed consent, and the study was approved by the respective institutional ethics committees.

**Table 1:** Baseline demographic and clinical characteristics of *BRCA* carriers, non-carriers, and healthy blood donors.

	*BRCA* carriers	Non-carriers	Healthy blood donors
*N*** total**	568	567	356
**Female sex,** *n* **(%)**	524 (92.3)	564 (99.5)	261 (73.3)
**Male sex,** *n* **(%)**	44 (7.7)	3 (0.5)	95 (26.7)
**Age, median (IQR), years**	51 (39–60)	52 (45–61)	29 (24–43)
**Breast cancer,** *n* **(%)**	324 (57.0)	567 (100)	NA
**Chemotherapy among BC cases,** *n* **(%)**	210 (64.8)	272 (48.0)	NA
***JAK2***^**V617F**^**+,** *n* **(%)**	24 (4.2)	24 (4.2)	6 (1.7)
**VAF, median (IQR), %**	0.062 (0.053–0.188)	0.069 (0.045–0.128)	0.091 (0.037–0.161)

Data are presented as *n* (%) for categorical variables and median (interquartile range, IQR) for continuous variables. Chemotherapy rates are calculated among individuals with breast cancer.

*NA* indicates not applicable, *BC* breast cancer, *VAF* variant allele frequency.

Genomic DNA was extracted from peripheral blood, and *JAK2*^V617F^ allele burden was quantified using allele-specific real-time quantitative PCR. Samples were considered positive if at least two of three total reactions yielded a signal. Analytical sensitivity was defined by the determination of the limit of blank and the limit of detection. Detailed assay characteristics are provided in the Supplementary Methods.

Statistical analyses were performed using GraphPad Prism (version 8.02, GraphPad Software, San Diego, CA, USA). Categorical variables were compared using Fisher’s exact test and continuous variables using the Mann–Whitney U test. Correlations were assessed using Spearman’s rank analysis. Odds ratios (OR) with 95% confidence intervals (CI) were calculated where appropriate. All tests were two-sided, and *p* < 0.05 was considered statistically significant.

## Results

**Fig. 1:**
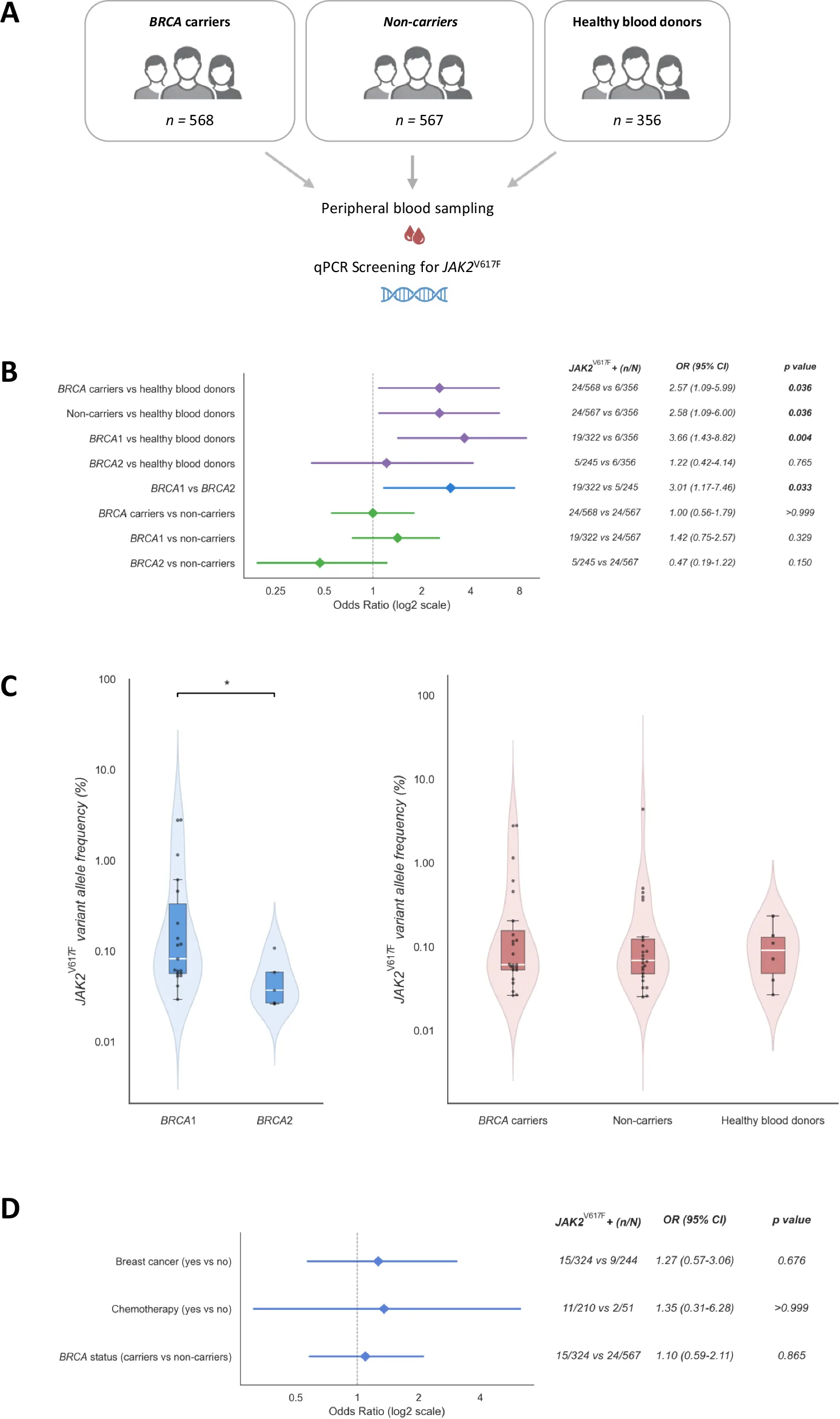
*JAK2*^V617F^ prevalence and allele burden according to *BRCA* mutation status and clinical variables. **A** Study overview. *BRCA* carriers, non-carriers, and healthy blood donors underwent peripheral blood sampling followed by allele-specific qPCR screening for *JAK2*^V617F^. **B** Frequency of *JAK2*^V617F^-positive individuals across study groups. Odds ratios (OR) with 95% confidence intervals (CI) were calculated from contingency tables using Fisher’s exact test. **C** *JAK2*^V617F^ variant allele frequency (VAF) among mutation-positive individuals is shown on a logarithmic scale. Left panel: VAF stratified by *BRCA1* versus *BRCA2* mutation status (Mann-Whitney U test). Right panel: VAF compared across *BRCA* carriers, non-carriers, and healthy blood donors (Kruskal–Wallis test). Each dot represents one individual. Violin plots depict the distribution of values; box plots indicate the median and interquartile range. **D** Association of *JAK2*^V617F^ positivity with clinical and genetic variables. Odds ratios (OR) with 95% confidence intervals (CI) are shown for breast cancer status (yes vs no) and chemotherapy exposure (yes vs no) among *BRCA* carriers, as well as for *BRCA* carrier status (carriers vs non-carriers) among breast cancer cases. ORs were calculated from contingency tables using Fisher’s exact test.

A total of 568 germline *BRCA1/2* mutation carriers (*BRCA* carriers), 567 *BRCA*-wildtype breast cancer patients (non-carriers), and 356 healthy blood donors were included (Table 1 and Fig. 1A; Supplementary Table 1). *BRCA* carriers and non-carriers were of comparable age (median 51 [IQR 39–60] vs 52 [45–61] years), whereas healthy donors were younger (29 [24–43] years) (Supplementary Fig. 1A). Consistent with the clinical context of *BRCA* testing, 57% of *BRCA* carriers and all non-carriers had breast cancer, and chemotherapy exposure was reported in 65% and 48%, respectively. The study population was predominantly female. One dual *BRCA1/2* carrier was included in overall analyses but excluded from subtype comparisons.

*JAK2*^V617F^ was detected in 24/568 *BRCA* carriers (4.2%) and 24/567 non-carriers (4.2%), compared with 6/356 healthy donors (1.7%) (Table 1). Compared with healthy blood donors, both *BRCA* carriers (24/568 vs 6/356) and non-carriers (24/567 vs 6/356) showed higher *JAK2*^V617F^ mutation frequencies (Fig. 1B). This difference between *BRCA* carriers and healthy blood donors was driven by *BRCA1* carriers (19/322 vs 6/356), whereas *BRCA2* carriers (5/245 vs 6/356) did not differ. Consistent with this, *BRCA1* carriers showed a higher mutation frequency than *BRCA2* carriers (19/322 [5.9%] vs 5/245 [2.0%]). In contrast, no difference was observed between *BRCA* carriers and non-carriers overall (24/568 vs 24/567), and neither *BRCA1* nor *BRCA2* carriers differed from non-carriers when analyzed separately (19/322 vs 24/567 and 5/245 vs 24/567, respectively). No sex-related differences were detected among *BRCA* carriers (2/44 males [4.5%] vs 22/524 females [4.2%]; *p* = 0.71; data not shown).

Among mutation-positive individuals, VAF was higher in *BRCA1* than *BRCA2* carriers (Fig. 1C, left). In contrast, when analyzed collectively, VAF did not differ between *BRCA* carriers, non-carriers, and healthy blood donors (Fig. 1C, right). Notably, descriptive stratification of allele burden categories revealed that VAF values ≥ 1% were more frequent in *BRCA1* carriers (3/19, 15.8%) than in non-carriers (1/24, 4.2%) and were absent in *BRCA2* carriers (0/5) (Supplementary Table 2). When applying a conventional CHIP threshold of VAF > 2%, this category included two *BRCA1* carriers, no *BRCA2* carriers, and one non-carrier.

To evaluate potential clinical confounders, *JAK2*^V617F^ positivity was analyzed according to breast cancer status and chemotherapy exposure (Fig. 1D). Among *BRCA* carriers, neither breast cancer history (15/324 vs 9/244) nor chemotherapy exposure (11/210 vs 2/51) was associated with *JAK2*^V617F^ positivity. Similarly, detection rates did not differ between *BRCA* carriers (15/324) and non-carriers (24/567) within breast cancer cases. In an exploratory pooled comparison of all breast cancer cases versus individuals without breast cancer (including healthy blood donors), no significant difference was observed (OR 1.79, 95% CI 0.98–3.31; *p* = 0.066, data not shown). Additionally, age was not associated with mutation detection or VAF within *BRCA* and non-carrier cohorts (Supplementary Fig. 1B, C).

## Discussion

Together, these findings indicate that *BRCA* subtype, rather than carrier status per se, influences *JAK2*^V617F^-positive clonal hematopoiesis. Although overall mutation frequencies were comparable between *BRCA* carriers and non-carriers, stratification revealed enrichment among *BRCA1* carriers, underscoring the importance of subtype-specific analyses in genetically predisposed populations. While global VAF distributions were similar across groups, the higher VAF categories observed in *BRCA1* carriers suggest subtype-specific differences; however, longitudinal sampling is needed to determine whether *BRCA1* status affects clone acquisition, expansion, or both.

Clinical and demographic variables did not account for these differences. Within the clinically applied breast cancer predisposition panel, additional pathogenic variants in genes other than *BRCA1/2* were identified in 3 of 24 *JAK2*^V617F^-positive *BRCA* carriers and in none of the 24 *JAK2*^V617F^-positive non-carriers, although undetected germline variants cannot be excluded.

A key limitation is the substantial age difference between patient cohorts and healthy blood donors, given the age dependency of clonal hematopoiesis. Although no age association was observed within *BRCA* and non-carrier cohorts, the small number of mutation-positive donors precluded robust age-adjusted comparisons, limiting interpretation of comparisons involving healthy donors. Nonetheless, the higher mutation frequency in breast cancer cohorts suggests that inflammatory or tumor-associated microenvironmental factors may contribute. Given that low-level *JAK2*^V617F^ positivity has been linked to systemic inflammation, tumor- or treatment-related selective pressures within the hematopoietic compartment may favor mutant clone persistence. These cell-extrinsic influences may also explain the similar aggregate prevalence observed between *BRCA* carriers and non-carriers, indicating that *JAK2*^V617F^ persistence is shaped not only by germline background but also by microenvironmental context.

BRCA1 and BRCA2 have partially distinct roles in homologous recombination. BRCA1 functions in early damage sensing and pathway regulation, whereas BRCA2 mediates RAD51 loading and strand invasion [12]. Heterozygous *BRCA1* loss may impair genome maintenance under replicative stress, increasing susceptibility to mutant clone emergence [13, 14]. Telomere dynamics may represent an additional layer, as BRCA1 participates in replication stress responses and telomere regulation; altered telomere maintenance has been linked to telomeric replication stress and genomic instability and could contribute to differential expansion of *JAK2*-mutant clones between *BRCA* subtypes.

These findings align with our prior work demonstrating enrichment of germline DNA repair variants in selected familial and young-onset MPN cohorts with a high prevalence of *JAK2*^V617F^ positivity [2]. Experimental models suggest that heterozygous DNA repair gene loss can exacerbate *JAK2*^V617F^-driven phenotypes and increase sensitivity to PARP inhibition, supporting a functional interaction between *BRCA1* haploinsufficiency and *JAK2*^V617F^-driven hematopoiesis [15]. PARP inhibitors, clinically used in *BRCA1/2*-mutated tumors based on synthetic lethality, increase replication-associated DNA damage in cells with impaired homologous recombination, and our results may provide a biological rationale for PARP inhibitor use in *BRCA1/2*-mutated MPN patients. Thus, *BRCA1*-haploinsufficient *JAK2*^V617F^-positive hematopoietic cells may show altered fitness under PARP inhibitor pressure.

Interpretation is limited by the small number of mutation-positive cases, particularly after subtype stratification, which restricted further treatment-stratified analyses. Data on chemotherapy classes and regimens were not uniformly available, and, therefore, the impacts of different chemotherapy regimens on clonal selection and the emergence of therapy-related clonal hematopoiesis could not be assessed. Because most *JAK2*^V617F^-positive clones had VAF < 2%, our findings mainly reflect low-VAF *JAK2*^V617F^-positive clonal hematopoiesis rather than conventional CHIP. The predominance of female participants reflects the clinical context of *BRCA* testing and limits sex-stratified analyses. Nonetheless, inclusion of a clinically matched *BRCA*-wildtype cohort alongside an unselected healthy donor population strengthens the subtype-specific conclusions.

From a translational perspective, these data highlight the importance of germline background in shaping *JAK2*^V617F^-positive CH. As *JAK2*^V617F^-positive CH represents a premalignant state that may precede myeloproliferative neoplasms, subtype-specific differences in clone burden could influence subsequent clonal dynamics. Although not designed to assess progression or therapeutic outcomes, the enrichment observed in *BRCA1* carriers suggests a biologically distinct context that may inform future risk stratification and therapeutic considerations in settings of DNA repair deficiency and *JAK2*-mutant hematopoiesis.

## Supplementary information

Supplementary Material

## Data Availability

De-identified participant-level clinical data and primary experimental data supporting the findings of this study are available from the corresponding author upon reasonable request and subject to institutional and ethical approval.
