# Supplementary material for: JAK2V617F-positive clonal hematopoiesis in germline BRCA1 versus BRCA2 mutation carriers
Source: Leukemia. 2026 Jul 15;40(9):2081–5. doi: 10.1038/s41375-026-03065-3 (PMC13506336; doi:10.1038/s41375-026-03065-3)

SUPPLEMENTAL INFORMATION

JAK2^{V617F}**-positive clonal hematopoiesis in germline *BRCA1* versus *BRCA2* mutation carriers**

Supplemental Methods

DNA Extraction

Genomic DNA was isolated from peripheral blood using the NucleoSpin Blood QuickPure kit (Macherey-Nagel, Düren, Germany) according to the manufacturer's instructions. DNA concentration and purity were assessed by spectrophotometry (NanoDrop, Thermo Fisher Scientific, Waltham, MA, USA), and only samples with an A260/280 ratio between 1.8 and 2.0 were included.

Calibration and Analytical Sensitivity of the *JAK2*^{V617F} qPCR Assay

A calibration curve was generated using genomic DNA derived from homozygous *JAK2*^{V617F} and wild-type human iPSC lines[1,2] mixed at defined ratios spanning allele burdens from 100% to 0%. The limit of blank (LoB), defined as the highest apparent signal expected in mutation-negative samples, corresponded to a predicted allele burden of 0.00028%. The limit of detection (LoD), defined as the lowest allele burden at which ≥95% of reactions yielded a positive signal, was 0.025%. Assay accuracy was validated using patient samples with previously quantified allele burdens (0.46%–98%) determined by next-generation sequencing.

PCR Conditions and Primer Sequences

PCR reactions were performed using 50 ng genomic DNA per reaction and allele-specific primers targeting the *JAK2*^{V617F}. Ct values were normalized using primers amplifying a wild-type *JAK2* region spanning exon-intron 7. Primer sequences (5′→3′) were as follows:

- *JAK2*^{V617F} allele-specific forward: TCCTCAGAACGTTGATGGCAG
- reverse: GTTTTACTTACTCTCGTCTCCACAAAA
- *JAK2* wild-type forward: CTGGAAACGGTGGAATTCAGTG
- reverse: CAAATGGGAGAAGTGCAATACC

Amplification specificity was verified by melt-curve analysis and agarose gel electrophoresis. Samples testing positive in the initial screening were reanalyzed in duplicate to confirm reproducibility.

Supplemental References

1 Satoh T, Toledo MAS, Boehnke J, Olschok K, Flosdorf N, Götz K *et al.* Human DC3 Antigen Presenting Dendritic Cells From Induced Pluripotent Stem Cells. *Front Cell Dev Biol* 2021; **9**: 667304.

2 Flosdorf N, Böhnke J, de Toledo MAS, Lutterbach N, Lerma VG, Graßhoff M *et al.* Proinflammatory phenotype of iPS cell-derived *JAK2* V617F megakaryocytes induces fibrosis in 3D in vitro bone marrow niche. *Stem Cell Reports* 2024; **19**: 224–238.

Supplemental Table 1. Baseline demographic and clinical characteristics of *BRCA1* and *BRCA2* carriers. Data are presented as n (%) for categorical variables and median (interquartile range, IQR) for continuous variables. Chemotherapy rates are calculated among individuals with breast cancer. BC indicates breast cancer; VAF, variant allele frequency; n, number.

	***BRCA1* carriers**	***BRCA2* carriers**
**N total**	322	245
**Female sex, n (%)**	299 (92.9)	224 (91.4)
**Male sex, n (%)**	23 (7.1)	21 (8.6)
**Age, median (IQR), years**	50 (38–59)	53 (41–63)
**Breast cancer, n (%)**	191 (59.3)	132 (53.9)
**Chemotherapy among BC cases, n (%)**	134 (70.2)	75 (56.8)
***JAK2*$^{\mathbf{V617F}}$+, n (%)**	19 (5.9)	5 (2.0)
**VAF, median (IQR), (%)**	0.083 (0.057–0.460)	0.037 (0.027–0.084)

Supplemental Table 2. Distribution of *JAK2*^{V617F} allele burden categories among mutation-positive individuals. *JAK2*^{V617F} variant allele frequency (VAF) was categorized into predefined allele burden ranges and summarized separately for *BRCA1* carriers, *BRCA2* carriers, and non-carriers. Data are presented as n (%), calculated within each group among *JAK2*^{V617F}-positive individuals.

**Allele burden category, VAF (%)**	***BRCA1* carriers (n = 19)**	***BRCA2* carriers (n = 5)**	**Non-carriers (n = 24)**
<0.1%	10 (52.6)	4 (80.0)	16 (66.7)
0.1–0.999%	6 (31.6)	1 (20.0)	7 (29.2)
≥1%	3 (15.8)	0 (0)	1 (4.2)

Supplemental Figure 1. Age distribution and age-related analyses of *JAK2*^{V617F} across study groups. (A) Age distribution of *BRCA* carriers, non-carriers, and healthy blood donors at the time of sample collection. Box-and-whisker plots display median age and interquartile range (IQR); whiskers indicate minimum to maximum values. (B) Age-stratified frequency of *JAK2*^{V617F} positivity in BRCA carriers and non-carriers. Bars represent the proportion of *JAK2*^{V617F}-positive individuals within each age category; numbers above bars indicate the total number of individuals per age group. (C) Association between age and *JAK2*^{V617F} variant allele frequency (VAF) among *JAK2*^{V617F}-positive individuals, shown separately for *BRCA* carriers and non-carriers. Each dot represents one individual; VAF is displayed on a logarithmic scale. Correlations were assessed using Spearman rank analysis.

A

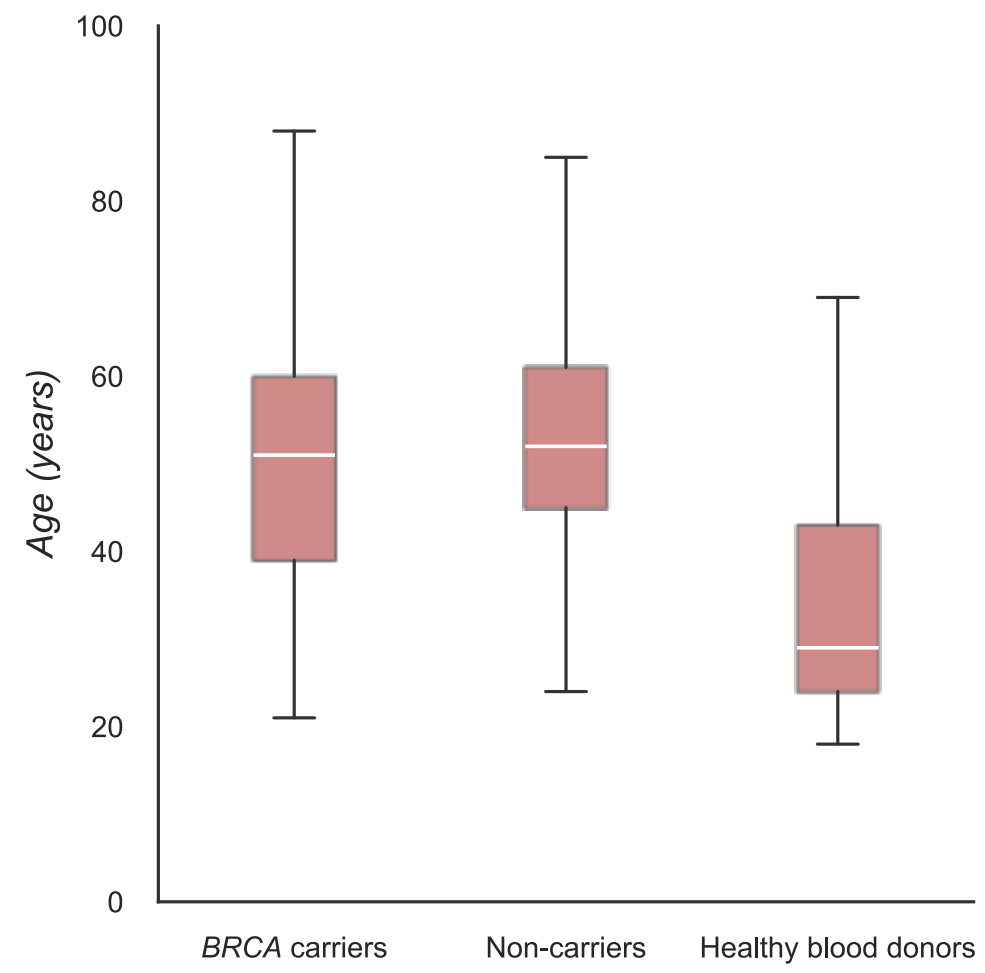

Study group	N	Median age (years)	IQR (years)
BRCA carriers	568	51	39–60
Non-carriers	567	52	45–61
Healthy blood donors	356	29	24–43

B

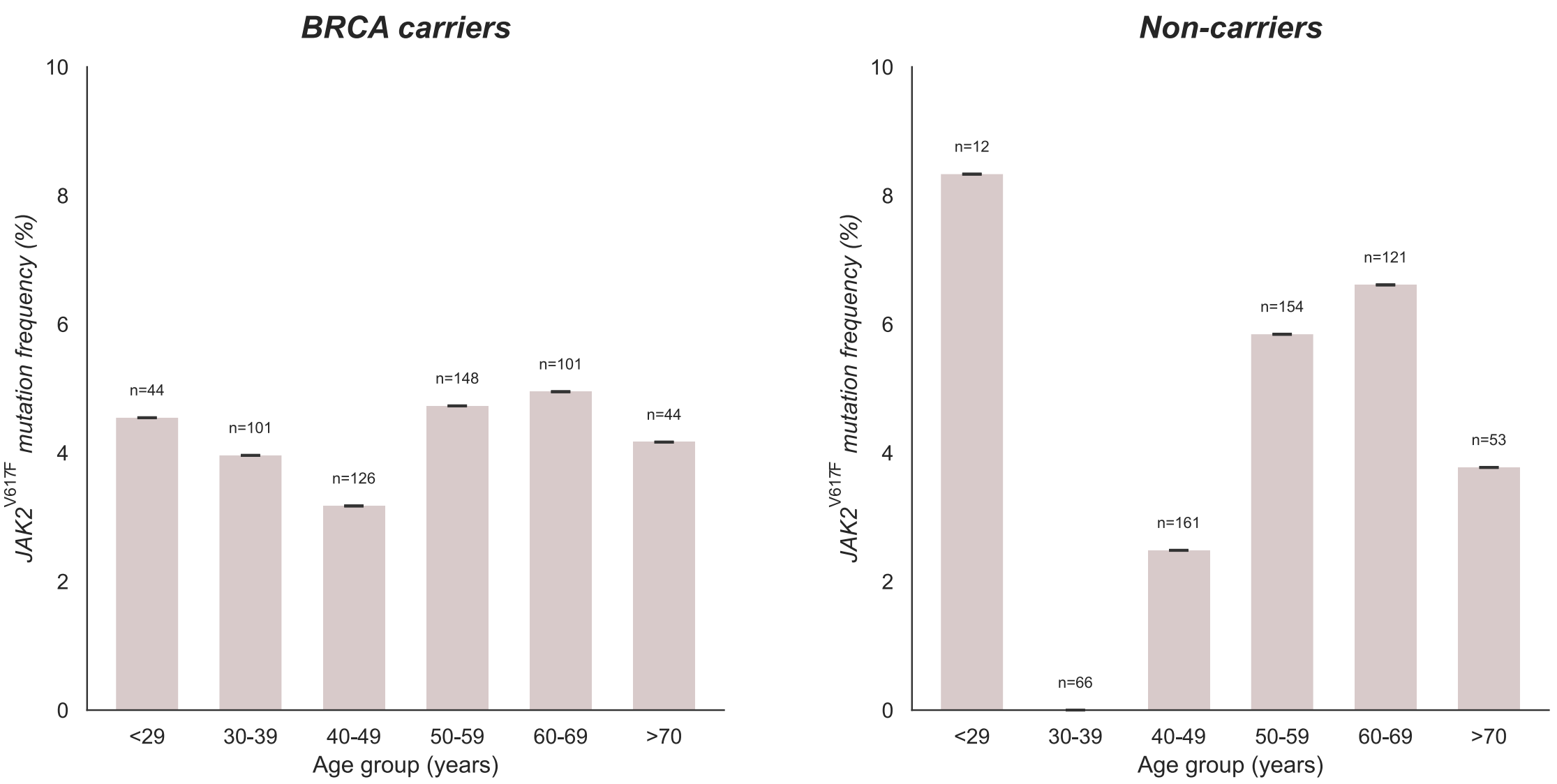

C

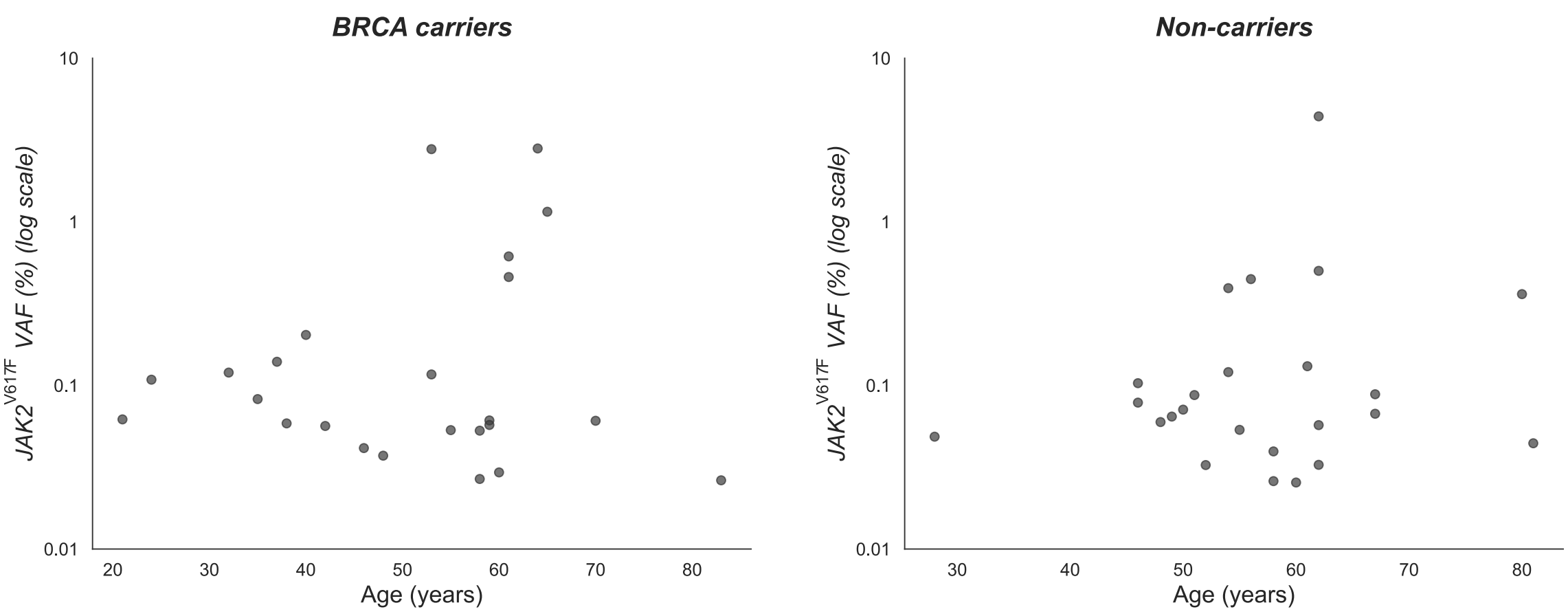

Supplement: Supplementary file 1 [file 41375_2026_3065_MOESM1_ESM.pdf]
